# Molecular biology of vascular bioenergetics rewiring: Mitochondrial protective mechanisms of *Piper retrofractum* in hypertension and oxidative injury

**DOI:** 10.1007/s11033-026-12366-w

**Published:** 2026-07-27

**Authors:** Anindini Winda Amalia, Liu Zhongyong, Huang Langlang, Reza Alrayan, Suci Nar Vikasari, Antonello Santini, Fahrul Nurkolis

**Affiliations:** 1 International Education College, Jiangxi University of Traditional Chinese Medicine, Nanchang, People’s Republic of China; 2 Department of Traditional Chinese Medicine, Faculty of Health, IIK Bhakti Wiyata Kediri, Kediri, Indonesia; 3 Department of Cardiology, Affiliated Hospital of Jiangxi University of Traditional Chinese Medicine, Nanchang, Jiangxi Province People’s Republic of China; 4 Department of Pharmacy, Faculty of Pharmacy, IIK Bhakti Wiyata Kediri, Kediri, Indonesia; 5 Department of Pharmacology and Clinical Pharmacy, Faculty of Pharmacy, Universitas Jenderal Achmad Yani, Cimahi, Indonesia; 6 Department of Pharmacy, University of Napoli Federico II, Via Domenico Montesano, 49, Napoli, 80131 Italy; 7 Medical Research Center of Indonesia, Surabaya, 60281 Indonesia; 8 Institute for Research and Community Service, State Islamic University of Sunan Kalijaga (UIN Sunan Kalijaga), Yogyakarta, 55281 Indonesia; 9 Faculty of Medicine, Universitas Airlangga, Surabaya, 60131 Indonesia

**Keywords:** *Piper retrofractum*, Hypertension, Mitochondrial dysfunction, Oxidative stress, Endothelial dysfunction, Nutraceuticals

## Abstract

Hypertension is a major contributor to cardiovascular morbidity and mortality, largely driven by oxidative stress, mitochondrial dysfunction, endothelial injury, and chronic vascular inflammation. Emerging evidence suggests that targeting vascular bioenergetics may provide complementary therapeutic strategies beyond conventional antihypertensive drugs. This review explores the mitochondrial protective mechanisms of *Piper retrofractum* and its major bioactive compounds in the context of hypertension and oxidative vascular injury. The phytochemical profile of *P. retrofractum*, particularly piperine, piplartine, pipernonaline, and retrofractamides, demonstrates significant antioxidant, anti-inflammatory, and metabolic regulatory activities. Mechanistically, these compounds may attenuate mitochondrial reactive oxygen species (mtROS), restore endothelial nitric oxide synthase (eNOS) coupling, activate AMPK–SIRT1–PGC-1α signaling, induce Nrf2–HO-1 antioxidant pathways, and modulate mitochondrial dynamics and mitophagy. Collectively, these effects contribute to improved endothelial function, reduced vascular remodeling, suppression of inflammatory cascades, and enhanced mitochondrial resilience. In addition, emerging omics technologies, network pharmacology, and AI-assisted nutraceutical discovery offer new opportunities to elucidate multitarget mechanisms and optimize *P. retrofractum*-based interventions. Despite promising preclinical evidence, important limitations remain, including insufficient clinical studies, limited mitochondrial-specific investigations, and challenges related to bioavailability and standardization. Overall, *P. retrofractum* represents a promising mitochondria-centered nutraceutical candidate for hypertension management and vascular protection, warranting further translational and clinical investigation.

## Introduction

### Global burden of hypertension

Hypertension affects roughly 1.4 billion adults worldwide (≈ 33% of people aged 30–79) [1]. It is a leading contributor to premature mortality, driving ~ 10.8 million deaths annually via cardiovascular and renal complications. Hypertension increases the risk of stroke, myocardial infarction, heart failure, renal failure and other vascular diseases [2, 3]. Despite the availability of antihypertensive drugs (ACE inhibitors, ARBs, β-blockers, etc.), many patients have uncontrolled blood pressure or residual vascular injury. Moreover, conventional therapies target systemic blood pressure but may not fully address the underlying oxidative and metabolic dysfunction in the vasculature. Thus, novel strategies that protect the vascular endothelium and its bioenergetics are needed to complement existing treatments.

### Oxidative stress as a central driver

Oxidative stress, an imbalance between pro-oxidants and antioxidants, is central to hypertension pathogenesis [4]. In hypertensive states, reactive oxygen species (ROS) such as superoxide (O₂⁻) and hydrogen peroxide (H₂O₂) are overproduced by multiple sources. Key contributors include dysfunctional mitochondria and activated NADPH oxidases (NOX1/2) in endothelial and smooth muscle cells. High levels of ROS oxidize lipids, proteins and DNA, damaging cells and activating inflammatory cascades. For example, Angiotensin II (AngII) stimulation of NOX1/2 in endothelial cells amplifies mitochondrial ROS production, leading to cytochrome c leakage and oxidative endothelial injury [5]. Excess ROS also inactivate nitric oxide (NO), promote formation of peroxynitrite, and oxidize tetrahydrobiopterin (BH₄), resulting in eNOS uncoupling [6]. Collectively, oxidative stress causes endothelial dysfunction, inflammation, and vascular remodeling (fibrosis, hypertrophy) that perpetuate hypertension. As Singh (2026) notes, hypertension models exhibit excessive mtROS and loss of NO bioavailability [7]. Inflammatory mediators such as NF-κB and the NLRP3 inflammasome are activated by mitochondrial damage, further driving vascular inflammation. Thus, breaking the cycle of oxidative stress is an attractive therapeutic goal in hypertension.

### Mitochondrial dysfunction in hypertensive disease

Mitochondria are cellular powerhouses and redox regulators in vascular cells. In hypertension, mitochondrial function is compromised: electron transport chain (ETC) activity is impaired, mitochondrial membrane potential (ΔΨm) is reduced, and Ca²⁺ homeostasis is disturbed [8]. These defects lead to excess mitochondrial ROS (mtROS) and diminished ATP production. For example, disruption of ETC Complex I leads to NAD⁺ depletion and burst of superoxide. Reduced ΔΨm and ATP impairs endothelial NO synthesis and barrier function [9]. Mitochondrial calcium overload via upregulated uniporters or pore opening (cyclophilin D, CypD) further depolarizes ΔΨm and triggers cell death [10]. Chronic mitochondrial damage (e.g. mtDNA mutations) accrues with age and hypertension, amplifying dysfunction. In fact, genomic analyses show hypertensive individuals harbor multiple mtDNA variants linked to cardiovascular complications [11]. Thus, dysfunctional mitochondria drive vascular aging: they produce toxic ROS, fail to supply energy for NO-mediated vasodilation, and release mtDNA and oxidized lipids that act as pro-inflammatory danger signals. Hypertension-induced mitochondrial injury also promotes *endothelial-to-mesenchymal transition* (EndoMT) and arterial stiffness [12]. In sum, mitochondrial dysregulation is a fundamental mechanism of hypertension and vascular disease.

### Emergence of mitochondrial nutraceuticals

Against this backdrop, there is growing interest in mitochondria-targeted nutraceuticals. Plant-derived bioactives (polyphenols, alkaloids, etc.) can modulate redox signaling and mitochondrial metabolism. By enhancing endogenous antioxidants or activating energy-sensing pathways, such phytochemicals may restore mitochondrial health. For instance, dietary compounds like resveratrol or EGCG have been shown to activate SIRT1/AMPK–PGC-1α and induce mitochondrial biogenesis [13, 14]. The concept of “mitochondrial nutraceuticals” posits that selectively improving mitochondrial function can ameliorate endothelial dysfunction and hypertension. Importantly, many traditional spices (e.g. *Piper*, *Curcuma*, *Ginkgo*) contain redox-active phytochemicals that could have such effects. However, systematic evaluations of their vascular mitochondrial actions are still limited.

### Herbal medicine and phytochemical strategies in hypertension

Growing interest in medicinal plants for hypertension management reflects increasing recognition that vascular dysfunction is driven not only by elevated blood pressure itself, but also by oxidative stress, endothelial injury, mitochondrial dysfunction, chronic inflammation, and vascular remodeling [15]. Conventional antihypertensive therapies remain highly effective for blood pressure control; however, residual vascular risk often persists, particularly in patients with endothelial dysfunction, metabolic syndrome, obesity, or oxidative vascular injury [16]. Consequently, complementary strategies involving plant-derived bioactives have gained substantial scientific attention as potential modulators of vascular homeostasis and mitochondrial resilience.

Numerous phytochemicals, including polyphenols, alkaloids, flavonoids, terpenoids, and phenolic acids, have demonstrated vascular-protective properties through mechanisms relevant to hypertension pathophysiology. These include enhancement of endothelial nitric oxide bioavailability, suppression of oxidative stress, modulation of inflammatory signaling pathways, attenuation of NADPH oxidase activity, and preservation of mitochondrial redox balance. Bioactive compounds such as resveratrol, epigallocatechin gallate (EGCG), quercetin, curcumin, and berberine have been investigated for their ability to improve endothelial function, reduce vascular stiffness, and modulate energy-sensing pathways including AMP-activated protein kinase (AMPK), sirtuin-1 (SIRT1), and nuclear factor erythroid 2–related factor 2 (Nrf2) [17, 18].

Importantly, medicinal plants may offer advantages beyond single-target pharmacology because many phytochemicals exhibit multitarget biological actions that simultaneously influence oxidative stress, inflammation, vascular metabolism, endothelial signaling, and mitochondrial homeostasis. This systems-level biological complexity is particularly relevant in hypertension, where vascular injury arises from interconnected pathophysiological mechanisms rather than isolated signaling abnormalities. Nevertheless, despite increasing interest in phytotherapeutics, translation into clinical hypertension management remains limited by variability in extract standardization, pharmacokinetic uncertainty, insufficient mechanistic validation, and limited high-quality human evidence. Within this broader context, *Piper retrofractum* emerges as a biologically interesting candidate because of its rich phytochemical profile, historical ethnomedicinal use, antioxidant and anti-inflammatory properties, and mechanistically plausible relevance to mitochondrial and endothelial biology. The following sections therefore focus specifically on whether *P. retrofractum* bioactives may plausibly contribute to mitochondrial protection in hypertensive vascular injury.

### Why Piper retrofractum?

*Piper retrofractum* Vahl (Javanese long pepper) is a Southeast Asian spice with a rich ethnopharmacology. Traditionally it is used as an anti-flatulent, expectorant and cough remedy in Indonesia and surrounding regions [19]. In Thai medicine, it is a key component of the “Trikha-tuk” herbal formula (with ginger and black pepper) known for promoting circulation and metabolic health. Modern phytochemistry shows *P. retrofractum* fruits and roots are rich in piperamides/alkaloids and phenolic amides. For example, piperine, piplartine (piperlongumine), pipernonaline, and unique “retrofractamides”. These compounds have documented bioactivities including antioxidant, anti-inflammatory and metabolic effects. For instance, pipernonaline and retrofractamides from *P. retrofractum* have been shown to modulate adipogenesis [20]. A recent review notes *P. retrofractum* extracts exhibit antioxidant, hepatoprotective, anti-obesity, and other protective effects [21]. Notably, a key study found that piperidine amides from *P. retrofractum* activated AMP-activated protein kinase (AMPK) and PPARδ, improving lipid metabolism and protecting against diet-induced obesity, hinting at potent metabolic reprogramming potential [22]. Despite this, little is known about the effects of *P. retrofractum* on vascular mitochondria and hypertension. Given its traditional use in circulatory remedies and its phytochemical profile, *P. retrofractum* is a promising candidate for a “mitochondrial nutraceutical” in vascular disease.

### Aim of the review

This review systematically examines how *Piper retrofractum* and its bioactive compounds could modulate mitochondrial and redox pathways in hypertensive vascular disease. We will first outline the phytochemistry and properties of *P. retrofractum* (Sect.  2), then detail the roles of mitochondria in vascular homeostasis and how they malfunction in hypertension (Sect.  3). We then focus on the mitochondria-protective mechanisms attributable to *P. retrofractum* (Sect.  4): these include direct antioxidant actions, activation of energy-sensing pathways (AMPK–SIRT1–PGC-1α), induction of endogenous antioxidant genes (Nrf2–HO-1), inhibition of NOX enzymes, restoration of eNOS coupling, and modulation of mitochondrial dynamics and quality control. Section  5 explores how these mitochondrial effects translate to organ-level protection in hypertensive oxidative injury (endothelial integrity, anti-fibrotic, reno/cardioprotective, etc.). In Sect.  6 we discuss omics and systems pharmacology approaches for multi-target drug discovery in this context. Section  7 considers translational aspects (nutraceutical development, nanoformulations, combination therapy, safety). Finally, we identify gaps (Sect.  8) and future directions (Sect.  9) for precision mitochondrial nutraceuticals in hypertension. Our goal is a comprehensive, mechanistic understanding to inspire novel research and therapeutic strategies leveraging *P. retrofractum*. Importantly, the central question guiding this review is whether *Piper retrofractum* bioactives may plausibly confer mitochondrial protection in hypertensive vascular injury through multitarget regulation of oxidative stress, endothelial dysfunction, inflammatory signaling, and bioenergetic homeostasis. Accordingly, mechanistic, translational, omics, and nutraceutical perspectives discussed throughout the manuscript are intended to support this central mitochondrial framework rather than represent independent thematic areas. Because mitochondrial dysfunction in hypertension is inherently multifactorial, a measured interdisciplinary perspective is considered necessary to contextualize the biological plausibility and translational potential of *P. retrofractum* within vascular bioenergetics.

### Literature search strategy and review scope

This manuscript was conducted as a narrative mechanistic review intended to synthesize current evidence regarding the potential mitochondrial-protective relevance of *Piper retrofractum* in hypertension and oxidative vascular injury. Unlike a systematic review, the purpose of this work was not to comprehensively identify all eligible studies through a predefined protocol, but rather to critically integrate mechanistic, pharmacological, and translational evidence relevant to vascular mitochondrial dysfunction. Literature searches were performed using electronic databases including PubMed, Scopus, Web of Science, and Google Scholar, focusing on publications available up to May 2026. Search terms included combinations of: “*Piper retrofractum*”, “piperine”, “piplartine”, “retrofractamide”, “hypertension”, “vascular dysfunction”, “mitochondria”, “mitochondrial oxidative stress”, “endothelial dysfunction”, “AMPK”, “Nrf2”, “oxidative stress”, “vascular inflammation”, and “nutraceutical”. Reference lists of relevant articles were also manually screened to identify additional studies of mechanistic or translational relevance. Study selection emphasized original research articles, mechanistic studies, preclinical experiments, translational pharmacology studies, and relevant reviews that informed the biological plausibility of *Piper retrofractum* in vascular mitochondrial protection. Studies involving related *Piper* species or isolated phytochemicals were included when direct *P. retrofractum* evidence was unavailable but mechanistically informative.

## Phytochemical landscape of *Piper retrofractum*

### Botanical characteristics

*Piper retrofractum* Vahl is a perennial climber in the Piperaceae family. It is closely related to *P. longum* (Indian long pepper) and *P. chaba*; some sources use “*Piper chaba*” as a synonym of *P. retrofractum* [23]. Native to Indonesia, Malaysia, and other parts of Southeast Asia, it thrives in tropical lowlands. The plant produces club-like fruit spikes (similar to black pepper but elongated) containing numerous ripe red peppercorns. In traditional practice, the fruit spikes are harvested, sun-dried and used whole or ground as a pungent spice and medicine. Ethnobotanically, *P. retrofractum* has been valued for digestive ailments (carminative, stomachic), respiratory conditions (expectorant, antitussive) and even as an aphrodisiac [24]. Its long history of culinary and medicinal use suggests a generally acceptable safety profile at culinary doses, although formal toxicology data on isolated compounds remain sparse. Geographically, major cultivation occurs in Indonesia (Java, Sumatra), Thailand, and Indochina, often intercropped or as an understory spice.

### Major bioactive compounds

Phytochemical studies reveal that *Piper retrofractum* is rich in piperamides (alkaloidal amides) and related phenolic compounds (Table 1). The major constituents include piperine, a well-known alkaloid responsible for the pungency of peppers and a major constituent of the fruit; piplartine (piperlongumine), an unsaturated amide recognized for its anticancer and anti-inflammatory properties; and several other amide alkaloids such as pipernonaline, piperlonguminine, guineensine, and chavicine, which are commonly found in long peppers. Unique compounds identified in *P. retrofractum* include retrofractamides A and B, alkylglycoside amides that differ structurally from piperine through glycosidic linkages. In addition, the plant contains minor amounts of phenylpropanoids and lignans, including dihydrobenzofuran lignans and flavonoids that contribute antioxidant activity, as well as terpenoids such as β-caryophyllene in the essential oil fraction. Other constituents include alkyl glycosides such as methyl piperate and various phenolic acids including caffeic and ferulic acids. Overall, the dominant phytochemicals in *P. retrofractum* are lipophilic amide alkaloids and phenolic amides that exhibit significant biological activities.

**Table 1:** Representative phytochemicals identified in *P. retrofractum* and their known bioactivities or molecular targets

Compound	Class	Notable mechanisms / Targets
Piperine (fruit)	Piperamide alkaloid	Activates AMPK (enhances fat metabolism) [25]; inhibits P-glycoprotein to improve drug and nutrient uptake [26]; antioxidant; vasodilatory activity through Ca²⁺ channel blockade [27]; anti-inflammatory via NF-κB inhibition [28].
Piplartine (Piperlongumine)	Piperamide alkaloid	Potent anti-inflammatory and pro-apoptotic agent; induces HO-1 expression and inhibits NF-κB signaling; exhibits anticancer effects in various cell lines [29, 30].
Retrofractamide A/B	Alkylglycoside amides	Modulates adipogenesis by inhibiting lipid accumulation in 3T3-L1 cells; potential activators of the Nrf2 pathway [31].
Pipernonaline (fruit)	Piperamide alkaloid	Exhibits antioxidant and anti-infective activities [32].
Guineensine (fruit)	Piperamide alkaloid	Inhibits anandamide reuptake, thereby potentiating endocannabinoid signaling; reported neuroactive and memory-enhancing effects in rodents [33, 34].
Methyl piperate (fruit)	Alkaloid	Identified in *P. retrofractum*; biological activities remain poorly characterized [35].
Flavonoids (minor compounds)	Phenolic compounds	Includes quercetin derivatives with antioxidant properties that may synergistically enhance alkaloid activity [36].

### Pharmacokinetic and physicochemical features

*Piper retrofractum* alkaloids are generally lipophilic and moderately sized, favoring membrane permeability. For example, piperine is a crystalline alkaloid (m.p. ~135 °C) with very low water solubility (~ 40 µg/mL) and a LogP around 2–3 [37]. These traits allow piperine and similar amides to readily cross cell membranes, including mitochondrial membranes, but they also result in poor aqueous absorption. Piperine is known to inhibit cytochrome P450 enzymes and P-glycoprotein (P-gp), thereby increasing bioavailability of co-administered compounds [38]. It undergoes extensive first-pass metabolism (glucuronidation and oxidation), yielding metabolites like piperonyl alcohol. As a result, oral bioavailability of piperine itself is modest, necessitating formulation strategies for therapeutic use. The other alkaloids (piplartine, pipernonaline) share similar lipophilicity and metabolic pathways (likely via hepatic enzymes) [39], though specific pharmacokinetics data are scarce for *P. retrofractum* extracts.

Mitochondrial targeting potential is notable. Highly lipophilic amides can accumulate in organelle membranes. Piperine has been used as a bioenhancer in drug delivery formulations (e.g. nanoemulsions, liposomes) to improve distribution and mitochondrial uptake [40]. However, their physicochemical properties also pose bioavailability issues; they are insoluble in water and can bind strongly to proteins. Recent studies emphasize formulating piperine (and analogs) into nanoparticles or inclusion complexes to enhance solubility and tissue delivery.

### Nutraceutical and functional food potential

From a nutraceutical perspective, *P. retrofractum* fruit extract is generally regarded as safe at culinary doses (as it is used in spices) and appears to have a broad safety margin. Toxicological data are limited, but preclinical studies have not reported severe toxicity at typical doses. The bioactive extracts also show antioxidant and anti-inflammatory effects in various models without overt cytotoxicity [41]. This supports its candidacy for dietary supplements or functional foods. As a spice, it could be standardized into capsules or extracts with quantified piperine/piperamides. Its pungent taste, however, may limit direct dietary use; encapsulation or flavor-masking might be needed in formulations. The recognized medicinal uses of related pepper species (e.g. *P. nigrum*, *P. longum*) in Ayurvedic and traditional Chinese medicine also endorse safety, although concentrated extracts should be evaluated in clinical trials [42]. In summary, *P. retrofractum* offers a favorable safety profile and versatility for nutraceutical development, pending further pharmacokinetic optimization.

## Vascular Bioenergetics and Mitochondrial Dysfunction in Hypertension

### Mitochondria in vascular homeostasis

Endothelial cells (ECs) and vascular smooth muscle cells (VSMCs) rely on mitochondria not only for ATP production but also for signaling. In ECs, despite their high glycolytic flux, mitochondria still supply ~ 20% of ATP used in basal conditions [43]. This mitochondrial ATP is crucial for processes like eNOS activation and Ca²⁺ handling that drive vasodilation. As Singh (2026) emphasizes, mitochondrial respiration (via complexes I–V) is essential for maintaining endothelial homeostasis (NO production and barrier integrity) even if glycolysis dominates energy production [7]. Moreover, mitochondria generate low “signaling” levels of ROS (mtROS) under physiological conditions. For example, in VEGF-induced angiogenesis, moderate superoxide or H₂O₂ acts as second messengers to promote endothelial proliferation and migration [44]. Mitochondria also regulate Ca²⁺ dynamics in VSMCs and ECs via the mitochondrial Ca²⁺ uniporter, influencing tone and barrier function. In summary, healthy vascular mitochondria provide the bioenergetic and redox signaling environment necessary for vasorelaxation, permeability, angiogenesis and resistance to stress. Disruption of these functions sensitizes vessels to dysfunction.

### Mitochondrial ROS in hypertension

Under hypertensive stimuli (AngII, shear stress, hyperglycemia), mitochondrial ROS production skyrockets. The principal ROS are superoxide (from electron leak in ETC) and its derivative H₂O₂. Singh (2026) notes that most studies of hypertension link excessive mtROS (O₂•⁻ and H₂O₂) to endothelial dysfunction [7]. These ROS directly damage endothelial cells: oxidizing lipids and proteins, inactivating NO, and oxidizing BH₄ leading to further ROS production by uncoupled eNOS. Genetic models underscore this: mice with constitutively acetylated SOD2 (mitochondrial superoxide dismutase) developed hypertension and vascular oxidative stress, whereas mice with SOD2 deacetylation (K68R) were protected [45]. AngII infusion in wild-type mice raised blood pressure and mtROS, but in NOX2-deficient mice this effect was blunted, showing crosstalk between NOX and mitochondria [46]. Thus, mtROS are both primary effectors of damage and amplified by parallel oxidase pathways. The net result is oxidative endothelial injury: reduced NO availability, increased peroxynitrite formation, and activation of redox-sensitive proinflammatory transcription factors (NF-κB). MtROS also contribute to vascular remodeling (see below). In short, unchecked mitochondrial ROS generation is a central driver of hypertension-induced vascular injury.

### Electron transport chain dysfunction

Hypertension impairs the ETC itself. Damage or downregulation of complexes I–V causes electron “leak” and ROS. For example, decreased NAD⁺ availability from complex I dysfunction raises mtROS levels. Frataxin deficiency (seen in hypertensive models) has been highlighted as a cause of ETC fragility: without frataxin, mitochondrial iron accumulates and promotes superoxide formation [47]. Consistent with this, hypertensive mice lacking frataxin showed exaggerated mtROS and vascular fibrosis (via EndoMT). In addition, impaired ETC causes energetic failure: reduced ATP and membrane potential (ΔΨm) [48]. Loss of ΔΨm in ECs activates pro-apoptotic cascades and barrier breakdown. In cardiomyocytes, ETC defects during hypertension contribute to contractile dysfunction. Overall, ETC dysfunction initiates a vicious cycle of oxidative stress and energy shortage in hypertensive vessels.

### eNOS uncoupling and NO depletion

Under oxidative conditions, endothelial nitric oxide synthase (eNOS) becomes uncoupled, a state in which instead of producing NO it generates superoxide. This occurs primarily because ROS oxidize BH₄ (a critical eNOS cofactor) to BH₂, depriving eNOS of substrate and forcing it to transfer electrons to O₂. The net effect is paradoxical: loss of NO (vasodilator, anti-inflammatory) and gain of superoxide. This mechanism compounds hypertension via reduced NO causes vasoconstriction and promotes inflammation, while eNOS-derived ROS adds to the overall oxidative burden [49, 50]. Although specific citations for eNOS uncoupling in *P. retrofractum* literature are lacking, this is a well-established mechanism in hypertension pathophysiology. Importantly, interventions that boost BH₄ or lower ROS can re-couple eNOS and restore NO signaling. For example, as noted above, promoting SOD2 activity preserved NO levels and prevented hypertension in mouse models [51]. Thus, one goal of antioxidant nutraceuticals is to prevent eNOS uncoupling and maintain NO bioavailability.

### Mitochondrial DNA damage

Mitochondrial DNA (mtDNA) is highly susceptible to oxidative damage. In hypertensive patients and models, oxidized mtDNA (e.g. isolevuglandin adducts) accumulates in vascular cells. These lesions impair mitochondrial gene expression and ETC assembly, further compromising respiration. In addition, mtDNA damage can trigger inflammatory responses: oxidized mtDNA fragments can activate the cytosolic cGAS–STING or NLRP3 pathways as “danger signals” [52]. It was noted that oxidative stress leading to mtDNA damage contributes to endothelial dysfunction and inflammatory signaling [53]. Over time, damaged mtDNA accelerates vascular aging such ascapillary rarefaction, stiffening and impaired repair. Therefore, preserving mtDNA integrity is another aspect of maintaining vascular bioenergetics in hypertension.

### Vascular remodeling and arterial stiffness

**Fig. 1:**
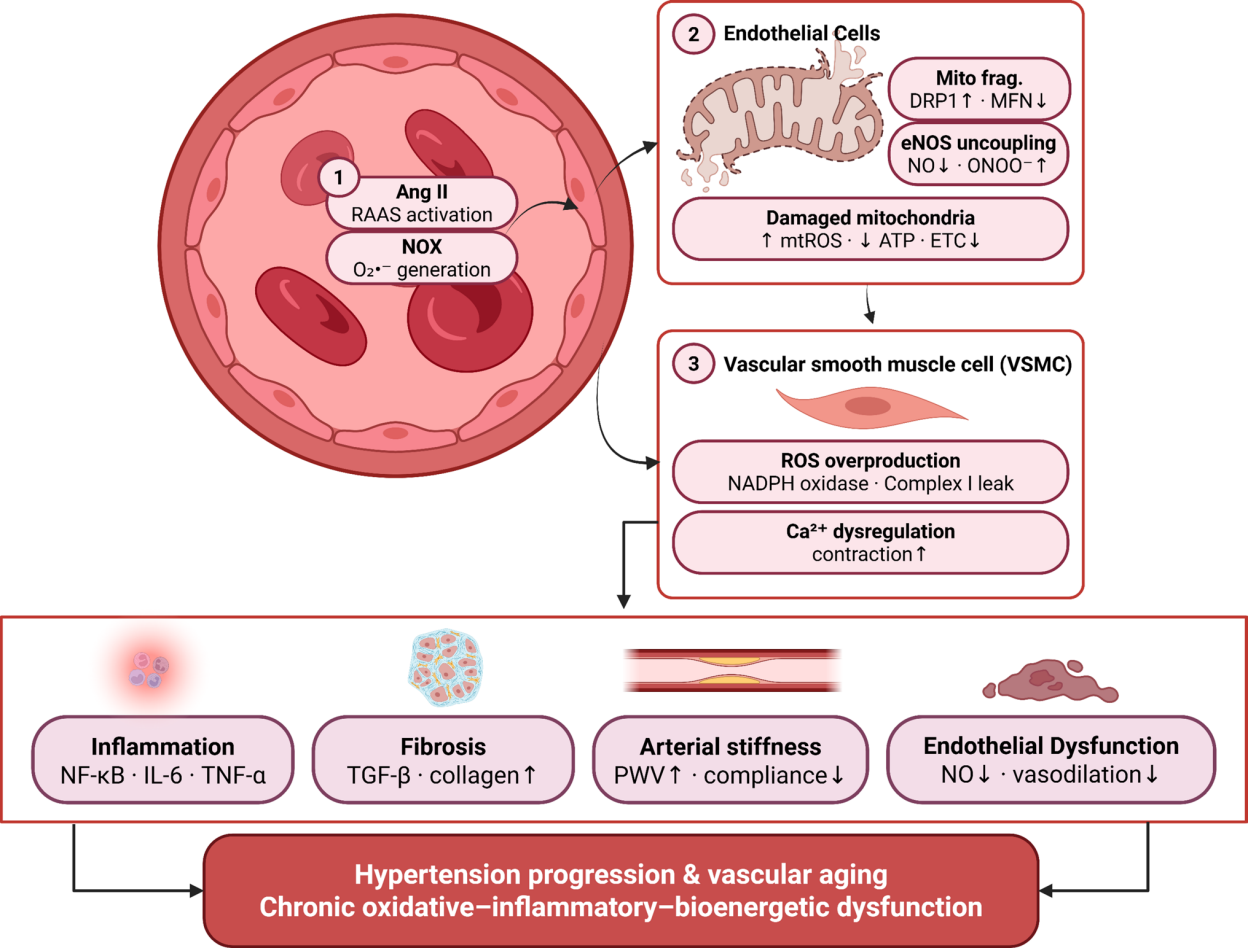
Mitochondrial dysfunction and oxidative injury in hypertensive vasculature. Hypertensive stimuli, including angiotensin II (Ang II) and NADPH oxidase (NOX) activation, promote excessive mitochondrial reactive oxygen species (mtROS) production and electron transport chain dysfunction in endothelial and vascular smooth muscle cells. Increased oxidative stress induces endothelial nitric oxide synthase (eNOS) uncoupling, nitric oxide (NO) depletion, mitochondrial DNA damage, ATP deficiency, and mitochondrial fragmentation. These bioenergetic disturbances amplify vascular inflammation, fibrosis, endothelial dysfunction, and arterial stiffness, ultimately contributing to hypertension progression and vascular aging. This figure was created using BioRender.com under a premium license by Fahrul Nurkolis

Chronic mitochondrial dysfunction and oxidative stress drive structural changes in vessels (Fig. 1). Endothelial injury and death provoke intimal thickening, while smooth muscle cells respond to ROS and cytokines by proliferating and migrating. Notably, Endothelial-to-Mesenchymal Transition (EndoMT), wherein ECs acquire fibrotic characteristics, is promoted by mitochondrial ROS [54]. In hypertensive models, loss of frataxin (with ensuing ROS) directly promoted EndoMT and vascular fibrosis [48]. At the same time, adventitial fibroblasts and VSMCs deposit collagen in response to cytokines (TGF-β, IL-6) that are upregulated by oxidative stress [55]. The end result is reduced arterial compliance and higher pulse pressure, which in turn perpetuates endothelial stress. Ultimately, “vascular aging” (a combination of remodeling and dysfunction) ensues, closing the loop between mitochondrial damage and hypertension.

## Mitochondrial protective mechanisms of *Piper retrofractum*

**Fig. 2:**
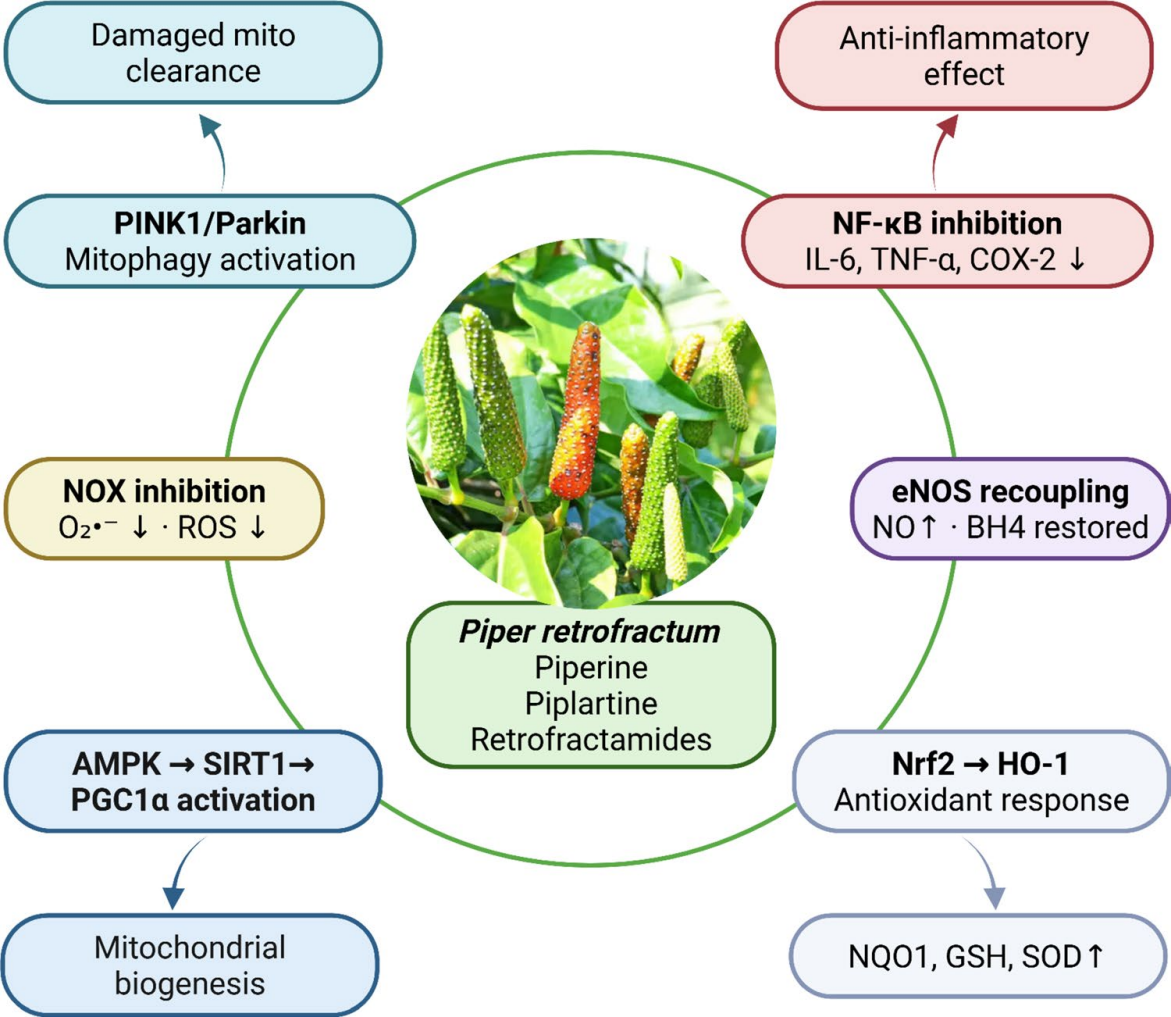
Molecular protective mechanisms of Piper retrofractum against mitochondrial oxidative vascular injury. Bioactive compounds derived from Piper retrofractum, including piperine, piplartine, and retrofractamides, exert multitarget protective actions through activation of AMPK/SIRT1/PGC1α-mediated mitochondrial biogenesis, induction of Nrf2/HO-1 antioxidant signaling, inhibition of NADPH oxidases (NOX), restoration of endothelial nitric oxide synthase (eNOS) coupling, and stimulation of PINK1/Parkin-dependent mitophagy. These mechanisms collectively improve mitochondrial quality control, reduce oxidative stress, suppress inflammation, and preserve endothelial bioenergetics. Disclaimer: This conceptual framework includes both comparatively stronger mechanistic evidence and biologically plausible inferred pathways; therefore, not all illustrated interactions should be interpreted as experimentally established actions of Piper retrofractum in hypertensive vascular tissues. This figure was created using BioRender.com under a premium license by Fahrul Nurkolis

Given the centrality of mitochondrial dysfunction in hypertension, *Piper retrofractum* phytochemicals could intervene at multiple points (Fig. 2). Here, we discuss putative mitochondria-protective mechanisms of *P. retrofractum* components, based on known actions of piperine and related compounds in cellular and animal studies (Table 2).

### Redox reprogramming and mtROS suppression

A primary effect of many plant alkaloids is to bolster antioxidant defenses. Piperine and piplartine possess phenolic or conjugated double bond structures that can scavenge free radicals, albeit less directly than flavonoids [56]. More importantly, *P. retrofractum* compounds may induce endogenous antioxidant pathways. For example, piperine has been shown to increase intracellular glutathione and antioxidant enzyme expression in some models [57]. This “redox reprogramming” shifts the cellular balance away from oxidative stress. Additionally, by stabilizing mitochondrial membranes, these compounds may reduce electron leak. Laboratory studies in other contexts suggest that Piper extracts decrease mitochondrial superoxide production under stress conditions [58]. While direct data on *P. retrofractum* is scant, its related species show similar trends. Thus, *P. retrofractum* bioactives are expected to scavenge mtROS and upregulate SOD2/GPx systems, breaking the feed-forward cycle of ROS-induced ROS release in mitochondria. Evidence Relevance, Current evidence supporting redox modulation by *Piper retrofractum* is derived primarily from antioxidant and anti-inflammatory studies in nonvascular systems or related Piper species. Although suppression of mitochondrial oxidative stress is mechanistically relevant to hypertensive vascular injury, direct evidence demonstrating attenuation of mitochondrial ROS in hypertensive endothelial tissues following *P. retrofractum* administration remains unavailable.

### AMPK–SIRT1–PGC1α Axis

The AMPK–SIRT1–PGC1α signaling axis is a master regulator of energy metabolism and mitochondrial biogenesis. Activation of AMPK (energy sensor kinase) increases NAD⁺ levels, activating SIRT1, which in turn deacetylates PGC-1α to drive mitochondrial gene expression and new mitochondria formation [59]. Intriguingly, K.J. Kim et al. (2011) demonstrated that piperidine amides from *P. retrofractum* activated AMPK in obese mice [22]. Piperidine amides isolated from *P. retrofractum* have been reported to activate AMPK signaling in obesity-related metabolic models, suggesting a mechanistically plausible role in mitochondrial biogenesis and metabolic regulation. However, these observations derive primarily from nonvascular systems and have not yet been biologically validated in hypertensive endothelial tissues. Upregulation of SIRT1 and PGC-1α would restore mitochondrial capacity and ATP production in endothelium. Enhanced mitochondrial turnover (biogenesis) could replace damaged organelles with fresh ones. In endothelium, this might normalize ATP homeostasis, making cells more resilient to stress. In summary, *P. retrofractum* may promote mitochondrial renewal via the AMPK–SIRT1–PGC1α pathway, countering the ATP depletion seen in hypertension. Evidence Relevance, The proposed role of *P. retrofractum* in AMPK–SIRT1–PGC-1α signaling is supported mainly by metabolic and obesity-related experimental models involving piperidine alkaloids rather than vascular systems. While activation of this pathway is biologically relevant to endothelial mitochondrial homeostasis, its direct involvement in hypertensive vascular mitochondria following *P. retrofractum* exposure has not yet been experimentally validated.

### Nrf2–HO-1 antioxidant signaling

Nuclear factor erythroid 2–related factor 2 (Nrf2) is a transcription factor that induces antioxidant gene programs when released from its inhibitor Keap1 [60]. Many phytochemicals activate Nrf2. While direct evidence for *P. retrofractum* is limited, piplartine and piperine analogs have been reported to induce Nrf2 [61]. Activation of Nrf2 increases expression of heme oxygenase-1 (HO-1), glutathione S-transferases and other cytoprotective genes. This enhances the cell’s ability to detoxify ROS and electrophiles. In vascular cells, Nrf2–HO-1 induction lowers oxidative stress and inflammatory signaling. Evidence derived from piplartine and structurally related piperamides suggests that *P. retrofractum* bioactives may influence Nrf2-mediated antioxidant signaling, promoting a cytoprotective antioxidant response. Such a mechanism would complement direct ROS scavenging and reinforce mitochondrial redox balance. Nevertheless, current support remains indirect and largely originates from nonvascular cellular systems, limiting conclusions regarding endothelial mitochondrial protection in hypertension. Evidence Relevance: Evidence supporting Nrf2–HO-1 modulation derives largely from studies involving piplartine, piperine analogs, or nonvascular cellular systems. Although these findings are mechanistically consistent with enhanced antioxidant defense and mitochondrial resilience, direct confirmation in hypertensive vascular endothelium remains lacking.

### NADPH oxidase (NOX) inhibition

NADPH oxidases are upstream sources of cellular ROS that can trigger mitochondrial oxidative injury. By inhibiting NOX isoforms, a compound can indirectly reduce mitochondrial ROS generation. Some polyphenols (resveratrol, apocynin) are known NOX inhibitors. In the case of piperine, direct NOX inhibition appears weak: Whitehouse et al. (2016) found piperine had negligible NOX-inhibitory effect in cell assays [62]. However, *P. retrofractum* extracts contain multiple amides beyond piperine, and the combination might suppress NOX gene expression. Even a partial reduction of NOX1/2/4 activity would attenuate the feed-forward ROS amplification. The Singh review notes that NOX2-deficient mice are protected from AngII-induced mtROS and hypertension [7]. By analogy, any NOX-suppressing action of *P. retrofractum* compounds could be vascular-protective. At minimum, their antioxidant actions would mitigate the downstream effects of any NOX-derived ROS. Evidence Relevance, Current evidence supporting NOX inhibition by *P. retrofractum* or its major compounds remains limited and somewhat inconsistent. Existing findings are largely indirect or derived from related phytochemicals rather than hypertensive vascular models. Therefore, any role in suppressing NOX-mediated mitochondrial oxidative injury should presently be interpreted as biologically plausible rather than experimentally established.

### Restoration of eNOS coupling

By lowering ROS and enhancing cofactors, *P. retrofractum* compounds may help recouple eNOS. Reduced oxidative stress preserves BH₄ and prevents eNOS from becoming a superoxide source. For instance, in hypertensive mouse models, enhancing mitochondrial SOD2 activity (via deacetylation mutation) increased endothelial NO levels and prevented blood pressure rise [63]. Similarly, by reducing mitochondrial and cytosolic ROS, *P. retrofractum* could restore NO bioavailability. Indeed, Piper extracts have been reported to relax blood vessels in vitro, likely via endothelium-dependent NO mechanisms [64]. Additionally, increased NO can stimulate mitochondrial biogenesis via cGMP, creating positive cross-talk. In summary, by suppressing oxidants that quench NO and preserving cofactors, *P. retrofractum* may promote endothelial relaxation. The net effect would be improved vascular tone regulation and antihypertensive action. Evidence Relevance, The proposed restoration of eNOS coupling is inferred primarily from the antioxidant and vasorelaxant properties of piperine and related Piper compounds rather than direct observations in hypertensive endothelial systems. While mechanistically relevant to vascular nitric oxide bioavailability, evidence demonstrating recoupling of eNOS by *P. retrofractum* in hypertension-specific vascular models is currently unavailable.

### Modulation of mitochondrial dynamics

Mitochondrial morphology is dynamically regulated by fusion (MFN1/2, OPA1) and fission (DRP1, FIS1). Hypertension tilts this balance toward excessive fission and fragmentation. Hypertensive stimuli upregulate DRP1 in ECs, leading to fragmented mitochondria, higher mtROS and depolarized ΔΨm [65]. Importantly, inhibiting DRP1 (preventing fission) normalized mitochondrial function in AngII-treated ECs. Thus, compounds that restore fusion or inhibit aberrant fission could protect mitochondrial networks. There is evidence that some phytochemicals modulate these pathways; for example, resveratrol was shown to increase MFN2 in muscle [66]. It is plausible that *P. retrofractum* amides affect fusion-fission proteins (though direct studies are lacking). At the very least, by reducing oxidative stress (which itself drives DRP1 activation), *P. retrofractum* indirectly favors healthy mitochondrial networks. At present, no direct evidence demonstrates that *P. retrofractum* modulates mitochondrial fusion–fission machinery in hypertensive vascular tissues. However, given the established relationship between oxidative stress and DRP1 activation, any antioxidant effects of *P. retrofractum* may plausibly influence mitochondrial dynamics indirectly. This possibility remains hypothetical and requires experimental validation. Evidence Relevance: At present, no direct evidence demonstrates that *P. retrofractum* modulates mitochondrial fusion–fission machinery (e.g., DRP1, MFN1/2, OPA1) in vascular or hypertensive tissues. The proposed effects on mitochondrial dynamics are therefore hypothesis-generating and extrapolated from broader oxidative stress literature and phytochemical mechanisms rather than experimentally verified observations.

### Mitophagy and cellular survival

Mitophagy is the selective autophagic clearance of damaged mitochondria, chiefly via the PINK1–Parkin pathway. Efficient mitophagy prevents accumulation of dysfunctional organelles. Compounds that activate mitophagy (e.g. by mild ROS activation of PINK1) can paradoxically be beneficial by removing the worst mitochondria. For example, the flavonoid dihydromyricetin promoted EC mitophagy and reduced oxidative stress in atherosclerosis models [67]. While direct evidence is lacking for *P. retrofractum*, it contains multiple bioactives that could induce mild mitochondrial stress (a “hormetic” effect) and trigger mitophagy. By clearing oxidized mitochondria, *P. retrofractum* would limit inflammatory signaling from damaged mtDNA and improve mitochondrial quality. Thus, enhanced mitophagy is a plausible mechanism: *P. retrofractum* fosters endothelial survival under hypertension by facilitating turnover of organelles. Evidence Relevance, The potential involvement of *P. retrofractum* in mitophagy regulation remains speculative and is inferred from analogous findings involving other phytochemicals in endothelial or inflammatory models. Although enhancement of mitochondrial quality control is mechanistically relevant to vascular protection, activation of PINK1/Parkin-mediated mitophagy by *P. retrofractum* has not yet been biologically demonstrated in hypertensive vascular tissues.

### Anti-inflammatory mitochondrial crosstalk

Lastly, *P. retrofractum* exerts anti-inflammatory effects that intersect with mitochondrial stress. The NF-κB pathway and NLRP3 inflammasome are key mediators of vascular inflammation in hypertension, often fueled by mitochondrial ROS and mtDNA. A recent study by Lallo et al. (2023) showed that *P. retrofractum* extract dramatically suppressed NF-κB activation and downstream cytokines (IL-6, IL-1β, COX-2) in macrophages and keratinocytes [41]. By dampening the TLR4–NF-κB axis, *P. retrofractum* could break the link between mitochondrial DAMPs and inflammatory gene expression. Although this study was in skin cells, it implies that vascular macrophages or ECs could similarly benefit. Furthermore, reducing mitochondrial ROS (via the above mechanisms) would itself attenuate NLRP3 inflammasome activation. In sum, *P. retrofractum* likely exerts retrograde crosstalk: its mitochondrial protection lowers ROS, which in turn reduces NF-κB/NLRP3 signaling; concurrently, direct inhibition of these pathways by its phytochemicals curbs inflammation. This bidirectional mitigation of oxidative and inflammatory stress reinforces vascular protection in hypertension. Evidence Relevance, the anti-inflammatory effects discussed in this section are supported primarily by studies conducted in macrophages, keratinocytes, and inflammatory nonvascular systems. Although suppression of NF-κB and inflammatory mediators is mechanistically relevant to oxidative vascular injury, the extent to which these findings translate to hypertensive vascular endothelium remains inferential and requires targeted investigation.

**Table 2:** Evidence relevance and confidence of proposed mitochondrial protective mechanisms of *Piper retrofractum* in hypertension

Proposed mechanism	Evidence source	Experimental model	Endpoint measured	Relevance to hypertensive vascular mitochondria	Confidence level
AMPK–SIRT1–PGC-1α activation	*P. retrofractum* piperidine alkaloids [18,21,55]	High-fat diet obese mice	AMPK activation, lipid metabolism	Mechanistically relevant, but not vascular-specific	Moderate
NF-κB suppression	*P. retrofractum* extract [25]	Keratinocytes or macrophages (skin inflammation model)	IL-6, IL-1β, COX-2, NF-κB activity	Anti-inflammatory relevance, but not vascular endothelium	Low–Moderate
Vasorelaxation or NO signaling	Piperine or related *Piper* species [65]	Coronary artery and vascular models	Vasorelaxation, endothelial function	More directly vascular, but species-specific	Moderate
Nrf2–HO-1 signaling	Piperine or piplartine-related studies [25,26,56,57]	Nonvascular cellular models	HO-1 induction, antioxidant signaling	Mechanistically plausible, not endothelially validated	Low
NOX inhibition	Piperine-related evidence [7,42,58]	Cell-based oxidative stress assays	NOX activity, ROS	Weak or inconsistent direct evidence	Low
Mitochondrial dynamics (fusion or fission)	Extrapolated from phytochemical literature [61,62]	Non-*Piper retrofractum* systems	DRP1/MFN signaling	Mechanistic hypothesis only	Very Low
Mitophagy (PINK1 or Parkin)	Analogous phytochemical studies [63]	Endothelial/atherosclerosis models	Mitophagy markers	Mechanistically plausible but untested in *P. retrofractum*	Very Low

## Vascular and organ protection in hypertensive oxidative injury

**Fig. 3:**
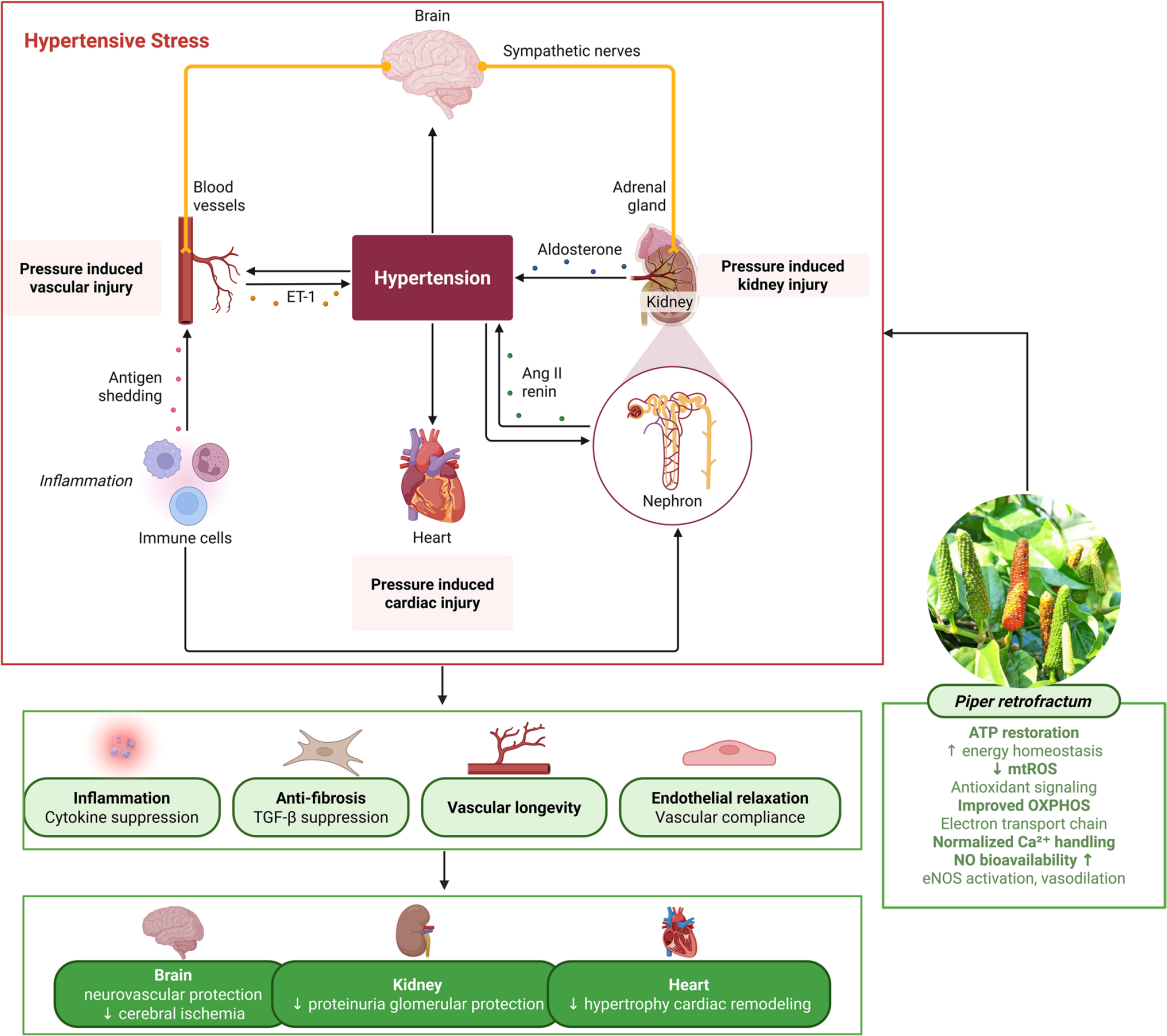
Integrated model of vascular bioenergetic rewiring induced by Piper retrofractum. Hypertensive oxidative stress disrupts mitochondrial respiration, enhances mtROS production, impairs nitric oxide signaling, and activates inflammatory pathways in vascular tissues. Piper retrofractum phytochemicals restore mitochondrial oxidative phosphorylation (OXPHOS), ATP homeostasis, endothelial nitric oxide bioavailability, and mitochondrial quality control while suppressing oxidative and inflammatory signaling cascades. These integrated effects improve endothelial function and confer vascular, renal, cardiac, and neurovascular protection. Disclaimer: Several protective pathways illustrated in this figure are mechanistically inferred from indirect evidence, related Piper species, or non-hypertension-specific models and should therefore be interpreted as biologically plausible rather than experimentally confirmed outcomes of Piper retrofractum. This figure was created using BioRender.com under a premium license by Fahrul Nurkolis

The mitochondrial and redox mechanisms described above translate into tangible protection of vascular and end-organ function (Table 3 and Fig. 3).

### Endothelial protection

Healthy endothelium depends on intact mitochondrial function. By preserving mitochondrial ATP and redox balance, *P. retrofractum* bioactives help maintain endothelial barrier integrity and anti-thrombotic properties. Reduced mtROS limits endothelial apoptosis and inflammatory adhesion. For instance, *P. retrofractum* treatment in inflamed tissues reduced endothelial damage markers and leukocyte infiltration (as evidenced in the skin inflammation model) [68]. In vessels, improved endothelial health would manifest as increased eNOS-derived NO and enhanced vasodilation. In vitro studies of Piper extracts (mainly piperine) have shown vasodilatory and anti-inflammatory effects in endothelial cells [69]. Thus, *P. retrofractum* is expected to protect the endothelium from hypertensive injury by sustaining mitochondrial energetics and blunting oxidative inflammation, preserving vascular permeability and function. Evidence Relevance: The endothelial-protective effects discussed in this section are supported primarily by anti-inflammatory studies involving *P. retrofractum* and vasorelaxant findings from related Piper species or isolated compounds such as piperine. While these observations are mechanistically relevant to endothelial dysfunction in hypertension, direct evidence demonstrating mitochondrial endothelial protection by *P. retrofractum* in hypertensive vascular systems remains limited.

### Anti-fibrotic effects

As noted, mitochondrial ROS drives vascular fibrosis via EndoMT and VSMC activation. By arresting this process at its root (mitochondrial dysfunction), *P. retrofractum* could attenuate fibrotic remodeling. In effect, reduced ROS and restored NO act as anti-fibrotic signals. There is some indirect support: other nutraceuticals that enhance SIRT3 or PGC-1α have been shown to lessen arterial stiffness [70]. In hypertensive mice, drugs that prevented mitochondrial calcium overload also reduced TGF-β signaling and collagen deposition. Although *P. retrofractum* studies have not directly measured collagen, its anti-inflammatory activity (↓ IL-1β, COX-2) and mitochondrial preservation suggest it would counteract pro-fibrotic signaling [41]. Overall, we predict *P. retrofractum* blunts the fibrogenic response in vessels by preventing the oxidative triggers of fibrosis. Evidence Relevance, the proposed anti-fibrotic effects of *P. retrofractum* are inferred from broader literature linking oxidative stress reduction and mitochondrial preservation to attenuation of fibrosis. However, no direct studies have evaluated whether *P. retrofractum* prevents vascular fibrosis or endothelial-to-mesenchymal transition in hypertensive models. Therefore, these effects should be considered biologically plausible but presently unvalidated.

### Renoprotective potential

The kidney is highly vulnerable to hypertension-induced oxidative damage. Hypertensive nephropathy involves glomerular injury, tubular damage, and maladaptive RAAS activation, all exacerbated by ROS. By boosting renal endothelial and tubular mitochondrial function, *P. retrofractum* may protect kidney architecture. For example, reduction of mtROS could mitigate glomerular endothelial dysfunction and preserve filtration. *P. retrofractum* extract’s known antioxidant effects (e.g. against drug-induced liver injury) imply renal benefits too [71]. Additionally, improved mitochondrial bioenergetics in renal cells might suppress ischemia-induced injury and inflammation. While specific data on *P. retrofractum* in renal models are lacking, its structural similarity to bioactive Piper species suggests renoprotective effects (e.g. via HO-1 induction or AT1R antagonism). In sum, enhancing mitochondrial resilience with *P. retrofractum* could slow hypertension-driven chronic kidney disease. Evidence Relevance, Evidence supporting renoprotective effects of *P. retrofractum* remains indirect and is derived largely from antioxidant or organ-protective studies unrelated to hypertensive nephropathy. Although mitochondrial oxidative stress is a shared pathological feature across organs, the relevance of these findings to hypertension-associated renal injury remains mechanistically suggestive rather than experimentally established.

### Cardioprotective effects

Hypertensive heart disease features left ventricular hypertrophy, myocardial fibrosis and diastolic dysfunction, processes tightly linked to mitochondrial stress in cardiomyocytes. SIRT3-deficient hypertensive mice exhibit marked cardiac mitochondrial swelling and fibrosis due to mPTP-mediated cell death [72]. By contrast, interventions that preserve SIRT3 and prevent mPTP opening improve cardiac outcomes. *P. retrofractum* components may increase SIRT1/3 activity (through AMPK/NAD⁺), thereby maintaining mitochondrial integrity in the heart. Moreover, reduced oxidative stress would inhibit hypertrophic signaling (e.g. via NF-κB). Experimental evidence shows that piperine can reduce infarct size in ischemia models by antioxidant actions [73]. Thus, *P. retrofractum* might protect the hypertensive myocardium by safeguarding mitochondria, limiting hypertrophic ROS signals and supporting efficient ATP supply. This could translate into lower risk of heart failure and arrhythmias in hypertension. Evidence Relevance: Cardioprotective findings associated with piperine and related Piper compounds derive mainly from ischemic or oxidative stress models rather than hypertensive cardiovascular disease. While preservation of mitochondrial integrity is mechanistically relevant to hypertensive cardiac remodeling, direct evidence supporting cardioprotective mitochondrial effects of *P. retrofractum* in hypertension-specific settings is currently lacking.

### Neurovascular protection

Hypertension is a major risk factor for cerebrovascular disease and cognitive decline. Cerebral small vessel disease and blood–brain barrier (BBB) disruption are aggravated by oxidative stress [74]. Mitochondrial dysfunction in cerebral endothelium impairs perfusion and leads to neuroinflammation. Although direct studies are limited, one may extrapolate: *P. retrofractum*’s endothelial-protective actions in systemic vessels should extend to cerebral microvessels. By reducing ROS and inflammation, *P. retrofractum* could help maintain BBB integrity and cerebral blood flow. Additionally, its anti-inflammatory cytokine suppression might benefit neurovascular coupling. Overall, *P. retrofractum* has potential to mitigate hypertension-associated cognitive impairment via its systemic vascular benefits, though specific trials will be needed. Evidence Relevance, the neurovascular implications discussed in this section are highly inferential and extrapolated from broader endothelial and oxidative stress literature rather than direct *P. retrofractum* evidence. Although mitochondrial dysfunction contributes to cerebrovascular complications in hypertension, the potential role of *P. retrofractum* in preserving neurovascular integrity remains hypothesis-generating and warrants dedicated investigation.

**Table 3:** Translational relevance of proposed vascular and organ-protective effects of *Piper retrofractum* in hypertension

Proposed protective effect	Evidence source	Experimental model	Tissue or system	Endpoint measured	Relevance to hypertensive vascular mitochondria	Confidence level
Endothelial protection	*P. retrofractum* extract; related *Piper* compounds [37,60,64,65]	Skin inflammation model; vascular studies in related species	Endothelium or inflammatory tissues	Reduced inflammatory markers, endothelial dysfunction, vasorelaxation	Mechanistically relevant but indirect	Moderate
Anti-fibrotic effects	Mitochondrial nutraceutical literature; indirect *Piper*-related evidence [44,50,51,66]	Hypertensive and oxidative stress models (non-*P. retrofractum*)	Vascular tissues	Reduced fibrosis signaling, oxidative stress	Biologically plausible but not directly tested	Low
Renoprotective potential	Antioxidant studies of *P. retrofractum* [67]	Hepatoprotective and oxidative stress animal models	Liver or systemic oxidative stress	Reduced oxidative injury and inflammation	Mechanistically informative, but kidney-specific evidence lacking	Low
Cardioprotective effects	Piperine-related studies [68,69]	Ischemia and oxidative injury models	Cardiac tissue	Reduced infarct size, oxidative stress	Mechanistically relevant but not hypertension-specific	Low–Moderate
Neurovascular protection	Broader oxidative stress and endothelial literature [70]	Neurological oxidative stress models	Cerebral vasculature or BBB	Reduced oxidative stress and inflammation	Hypothesis-generating and indirect	Very Low

Collectively, these vascular and organ-level observations suggest that the biological plausibility of *Piper retrofractum* in hypertension may lie not in a single dominant pathway, but rather in coordinated modulation of mitochondrial oxidative stress, endothelial dysfunction, inflammatory signaling, and vascular bioenergetic resilience. However, because much of the current evidence remains indirect or extrapolated from nonvascular systems, further mechanistic validation is required before translational conclusions can be drawn.

## Omics and systems pharmacology perspectives

Although the mechanistic pathways discussed in Sects.  4 and 5 provide biologically plausible explanations for how *Piper retrofractum* may influence vascular mitochondrial homeostasis, several proposed mechanisms remain incompletely validated in hypertension-specific systems. Accordingly, emerging systems-level approaches—including omics technologies, network pharmacology, and AI-assisted discovery—are discussed in this section not as independent themes, but as translational tools that may help experimentally validate mitochondrial mechanisms, identify bioactive targets, prioritize candidate compounds, and refine future precision nutraceutical development. Modern “-omics” and computational approaches offer tools to comprehensively map *P. retrofractum*’s multitarget actions.

### Metabolomics

Metabolomic profiling of *P. retrofractum* extracts and treated tissues can identify active compounds and metabolic shifts. For example, LC-MS/MS studies could reveal the concentration of piperamides (piperine, etc.) and their metabolites in plasma or organs. In parallel, assessing vascular metabolite signatures (NAD⁺/NADH ratios, glutathione, lipid peroxidation products) before and after treatment would clarify biochemical effects. Such profiling might show a “Warburg-like” shift (as reported in Tri-Ka-Tuk-treated models) and enhanced mitochondrial TCA cycle intermediates. Metabolomics of endothelial cells exposed to *P. retrofractum* could pinpoint upregulated antioxidants (e.g. GSH) or altered amino acid metabolism linked to NO production. Overall, metabolomics can track how *P. retrofractum* reprograms redox and energy pathways.

### Network pharmacology

Given the complex phytochemistry, *P. retrofractum* likely acts on many molecular targets. Network pharmacology (in silico construction of herb–compound–target networks) can predict interactions of its compounds with human proteins. Using databases and predictive algorithms, one could identify mitochondrial proteins (e.g. SIRT3, AMPK, PPARδ, eNOS) potentially modulated by the main alkaloids. Pathway enrichment analysis could highlight relevant signaling routes (AMPK, Nrf2, NF-κB, PI3K/Akt, etc.) intersecting with vascular redox. Such systems models would suggest synergies (e.g. piperine and piplartine both affecting NOX and inflammatory mediators). Network models have been used for other Piper species; applying them to *P. retrofractum* would prioritize mechanisms for experimental testing [75, 76].

### Molecular docking and dynamics

In silico docking can further support likely targets. For instance, molecular docking of piperine and piplartine against key mitochondrial enzymes (e.g. complex I subunits, PDK, CypD) or receptors (AT1R, ACE) may reveal binding affinities that explain observed effects [77]. Docking could also test whether *P. retrofractum* compounds fit in the active sites of SOD2 or catalase (enhancing their activity) or HO-1. Combined with molecular dynamics simulations, this approach can predict stability and potency of interactions. For example, if piperine binds Keap1, it could release Nrf2. Similarly, docking to kinases might show AMPK activation potential. These computational insights guide targeted experiments on the most promising molecular interactions.

### Multi-omics integration

The ultimate systems view integrates transcriptomics (gene expression), proteomics, and metabolomics. Transcriptomic profiling of vascular tissue or endothelial cells treated with *P. retrofractum* extract could reveal upregulation of mitochondrial biogenesis genes (PGC-1α, NRF1/2), antioxidant genes (HO-1, SOD2), and anti-inflammatory genes. Proteomics could confirm changes in protein levels or post-translational modifications (e.g. increased SIRT3 or decreased acetylated SOD2). Redox metabolomics (measuring specific ROS and redox cofactors) would add another dimension [78]. Integrating these data (e.g. via network/pathway analysis) can reconstruct the entire response to *P. retrofractum*, highlighting both primary targets and downstream effects on vascular homeostasis. While such studies are intensive, they could unequivocally demonstrate a mitochondrial-centric mode of action.

### AI-assisted nutraceutical discovery

Artificial intelligence and machine learning are emerging in nutraceutical research. Predictive algorithms can screen virtual libraries of Piper-related compounds for activity on mitochondrial targets. AI can also design novel analogs of piperine with improved mitochondrial penetration or selectivity. Furthermore, systems modeling could simulate patient-specific responses, leading to precision nutraceuticals tailored to genetic profiles (e.g. SIRT3 or NOX polymorphisms). Combining AI with omics data (e.g. training a model on *P. retrofractum* bioactivity datasets) may accelerate identification of optimal multi-component formulations. This frontier promises to transform *Piper* research from descriptive to predictive and personalized.

## Translational and clinical perspectives

Given that the proposed mitochondrial-protective actions of *Piper retrofractum* remain predominantly preclinical and mechanistically inferred, translational considerations are important to contextualize the feasibility of future therapeutic development. Therefore, the following discussion does not assume established clinical efficacy, but instead evaluates practical considerations—including pharmacokinetics, formulation challenges, safety, and bioavailability—that would need to be addressed before mitochondria-centered vascular applications of *P. retrofractum* could be realistically considered.

### Functional food and nutraceutical development

To translate these findings, *P. retrofractum* can be developed as a functional food or nutraceutical. Potential products include standardized dried fruit extracts, capsules rich in piperamides, or fortified herbal teas. Quality control would require quantification of marker compounds (e.g. piperine content, which in *P. retrofractum* is about 3–4%) [79]. Such extracts could be marketed for cardiovascular health, similar to other pepper supplements. Early human studies might focus on markers of oxidative stress and endothelial function (e.g. flow-mediated dilation) in hypertensive subjects. Encouragingly, black pepper (rich in piperine) has been safely consumed for millennia, suggesting a wide therapeutic window [80]. Regulatory approval for health claims would require clinical trials demonstrating blood pressure or vascular benefits.

### Translational pharmacology and clinical feasibility of Piper retrofractum

Despite promising mechanistic evidence, the translational feasibility of *Piper retrofractum* bioactives in hypertension remains constrained by pharmacokinetic and toxicological uncertainties (Table 4). Among its constituents, piperine is currently the best-characterized compound and demonstrates several favorable translational features, including lipophilicity and membrane permeability [33–36, 75]. However, oral bioavailability remains limited due to poor aqueous solubility and extensive first-pass metabolism involving glucuronidation and oxidative biotransformation [33–36, 75, 79]. Experimental studies indicate that piperine undergoes substantial hepatic metabolism, potentially limiting systemic concentrations despite gastrointestinal absorption [33–36]. Consequently, although preclinical findings support antioxidant and metabolic effects, whether vascular mitochondrial tissues in humans can achieve biologically effective concentrations remains uncertain [35, 75].

An important translational consideration involves enzyme and transporter interactions. Piperine is widely recognized as a bioenhancer due to its inhibitory effects on cytochrome P450 enzymes, including CYP3A4, CYP2C9, and CYP2D6, as well as P-glycoprotein (P-gp)-mediated drug efflux [33–36, 79]. These properties may increase systemic exposure of co-administered drugs and therefore introduce clinically relevant herb–drug interaction risks [35, 79]. In hypertensive populations, this issue warrants careful consideration because commonly prescribed antihypertensive agents—including calcium channel blockers, β-blockers, angiotensin receptor blockers, and certain diuretics—may share overlapping metabolic pathways. While synergistic therapeutic effects are theoretically possible, altered drug clearance or enhanced systemic exposure could also increase adverse-event risks [79]. In contrast, pharmacokinetic information regarding piplartine, retrofractamides, and pipernonaline remains limited, and their absorption, metabolism, tissue distribution, and elimination profiles are insufficiently characterized [25, 26, 35].

Dose translation also remains an important unresolved challenge. Most experimental evidence involving *Piper retrofractum* bioactives derives from in vitro studies or animal models employing concentrations that may not directly reflect achievable human exposure levels [25, 35, 75]. Although piperine is commercially available as a dietary supplement in doses generally ranging between 5 and 20 mg/day [33, 75], whether such exposure produces sufficient mitochondrial concentrations in vascular tissues to influence hypertensive pathophysiology remains unknown. Furthermore, phytochemical variability resulting from geographical origin, extraction method, and standardization practices may substantially alter pharmacological potency across preparations [37, 80].

Before clinical translation can be reasonably considered, several preclinical investigations remain necessary. These include comprehensive pharmacokinetic studies defining absorption, distribution, metabolism, and excretion (ADME) characteristics of major *Piper retrofractum* constituents; dose–response assessments in hypertension-specific animal models; vascular mitochondrial biodistribution studies; standardized extract characterization; and chronic toxicology evaluations [37, 75, 80]. Additional investigations assessing herb–drug interaction risks with commonly prescribed antihypertensive agents are also needed [79]. Therefore, while *Piper retrofractum* represents a promising preclinical nutraceutical candidate, human therapeutic claims remain premature pending rigorous translational validation.

**Table 4:** Translational pharmacology and clinical feasibility of major *Piper retrofractum* bioactives

Compound	Oral bioavailability (ADME)	Major metabolism	Enzyme / Transporter interaction	Dose range	Plausible human exposure	Key limitation
Piperine [33–36,75,79]	Low–moderate oral bioavailability due to poor aqueous solubility; lipophilic with efficient membrane permeability	Extensive first-pass hepatic metabolism (oxidation, glucuronidation)	Inhibits CYP3A4, CYP2D6, CYP2C9 and P-glycoprotein (P-gp)	~ 5–20 mg/kg in animal studies	Human supplement doses (~ 5–20 mg/day) may achieve systemic exposure, though vascular mitochondrial concentrations remain uncertain	High pharmacokinetic variability and drug–drug interaction potential
Piplartine (Piperlongumine) [25,26,35]	Limited pharmacokinetic data; lipophilic compound with presumed moderate membrane penetration	Likely hepatic biotransformation via oxidative metabolism	Potential CYP interactions suspected but insufficiently characterized	Typically 2–10 mg/kg in preclinical models	Human exposure feasibility remains uncertain due to lack of PK data	Insufficient human pharmacokinetic evidence
Pipernonaline / Retrofractamides	Pharmacokinetic profile poorly characterized	Unknown; likely hepatic metabolism	Unknown transporter interactions	Primarily in vitro evidence	Human translational feasibility remains highly uncertain	Lack of ADME and toxicological data
*P. retrofractum* extract [37,75,80]	Variable depending on extraction method and phytochemical standardization	Multi-compound metabolism	Possible synergistic CYP/P-gp effects due to piperine content	Experimental doses vary considerably	Standardized formulations would be required for reproducible clinical translation	Batch variability and dose standardization challenges

### Nanoformulation strategies

Given the lipophilicity and bioavailability issues of piperamides, nano-delivery systems will be crucial. As shown for piperine, nanoemulsions, solid lipid nanoparticles, liposomes and polymeric micelles can significantly enhance absorption and target mitochondria. For instance, piperine-loaded lipid nanoparticles improved cellular uptake and antioxidant efficacy in cell models [81]. Similarly, encapsulating a *P. retrofractum* extract in nanocarriers could ensure sustained release to endothelial cells. Specific mitochondria-targeted delivery (e.g. triphenylphosphonium-conjugated liposomes) might further boost efficacy. Such innovations would turn *P. retrofractum* into a more potent nutraceutical and could allow lower doses (minimizing any off-target effects). Development of a nanoformulated nutraceutical could be guided by the pharmacokinetic profiles of Piper compounds identified in Sect.  2.3.

### Combination therapy opportunities

*P. retrofractum* nutraceuticals could be used adjunctively with standard antihypertensives. For example, combining a Piper extract with an ACE inhibitor or ARB might achieve synergistic blood pressure lowering; the drug reduces systemic pressure while the nutraceutical protects the vasculature [82]. The multi-target nature of phytochemicals also allows combination with other antioxidants (vitamin C/E) or vasodilators (nitrates). However, caution is warranted regarding herb–drug interactions. For instance, piperine’s inhibition of CYP enzymes could alter metabolism of co-administered drugs [83]. Controlled studies are needed to identify beneficial vs. adverse interactions. Overall, *P. retrofractum* could fit into a multi-modal hypertension regimen, reinforcing mitochondrial and redox balance alongside conventional pharmacology.

### Safety and toxicological considerations

At dietary levels, *P. retrofractum* is expected to be safe. Preclinical studies should evaluate higher-dose toxicity: acute and chronic rodent studies measuring liver, kidney function and histology. A relevant safety marker is gastrointestinal tolerance, as high doses of piperine can irritate the gut. One toxicity study reported *P. retrofractum* infusions have low toxicity (LD₅₀ in mice ~ 2.1 g/kg) [84], placing it in a low-toxicity category. Nonetheless, standardized dosing is critical: active doses (e.g. piperine 10–20 mg/kg) must avoid cumulative toxicity. Women planning pregnancy should likely avoid concentrated pepper extracts. In smokers or patients on multiple drugs, caution is also needed due to metabolic interactions. Overall, *P. retrofractum* appears safe, but clinical trials must monitor safety endpoints, especially liver enzymes and blood counts.

## Current limitations and research gaps

Despite the promising therapeutic potential of *Piper retrofractum*, several important limitations and research gaps remain unresolved. One major challenge is the lack of mitochondria-specific investigations examining the direct effects of *P. retrofractum* on endothelial or vascular mitochondrial function under hypertensive conditions. Current evidence is largely indirect, derived either from related *Piper* species or from general pharmacological studies unrelated to vascular mitochondria. Consequently, targeted experimental studies are still needed, such as investigations using hypertensive animal models to evaluate mitochondrial respiration, oxidative stress, and endothelial responses following treatment with *P. retrofractum* extracts or isolated compounds.

Another critical limitation is the scarcity of clinical evidence. To date, no human clinical trials have specifically evaluated *P. retrofractum* for hypertension management or vascular mitochondrial protection. Most available findings originate from in vitro assays or animal studies focusing on broader anti-inflammatory, antioxidant, or metabolic effects. As a result, the translational relevance of these findings to human cardiovascular health remains uncertain. In addition, emerging vascular organoid and organ-on-chip technologies, which could provide physiologically relevant three-dimensional models for testing Piper-derived compounds, have not yet been widely applied in this field [85]. Such advanced models may help clarify complex tissue-level interactions and improve the predictive value of preclinical studies.

Research is also limited by the lack of integrated multi-omics approaches. Many mechanistic studies focus only on isolated signaling pathways, while comprehensive transcriptomic, proteomic, or metabolomic analyses in vascular tissues treated with *P. retrofractum* are still absent. This restricts a systems-level understanding of how Piper compounds influence mitochondrial and vascular biology. Furthermore, standardization remains a major challenge because phytochemical composition can vary depending on geographical origin, cultivation practices, harvest timing, and extraction methods. Currently, there is no universally standardized *P. retrofractum* preparation, making reproducibility across studies difficult. Future nutraceutical development will therefore require rigorous quality-control systems, including standardized quantification of major compounds such as piperine and piplartine.

Additional concerns involve bioavailability, dosing, and safety. Effective human dosages have not been clearly established, and pharmacologically active concentrations may exceed those achievable through normal dietary consumption. Higher doses could also increase the risk of adverse effects. Although strategies such as nanoformulations or carrier-based delivery systems may improve bioavailability, their long-term safety, efficacy, and economic feasibility remain to be validated. Moreover, the pleiotropic nature of Piper compounds raises the possibility of off-target pharmacological effects, including interactions involving TRPV1 signaling, gastrointestinal motility, or other physiological pathways [86]. Comprehensive profiling of these secondary activities is therefore necessary.

Finally, hypertension itself is a highly heterogeneous disease influenced by genetic, metabolic, environmental, and dietary factors. It remains unclear which patient populations would benefit most from mitochondrial-targeted nutraceutical interventions derived from *P. retrofractum*. Addressing these limitations will require multidisciplinary collaboration integrating pharmacology, cardiovascular physiology, molecular biology, toxicology, bioinformatics, and formulation science to establish both mechanistic clarity and clinical relevance.

## Future perspectives

**Fig. 4:**
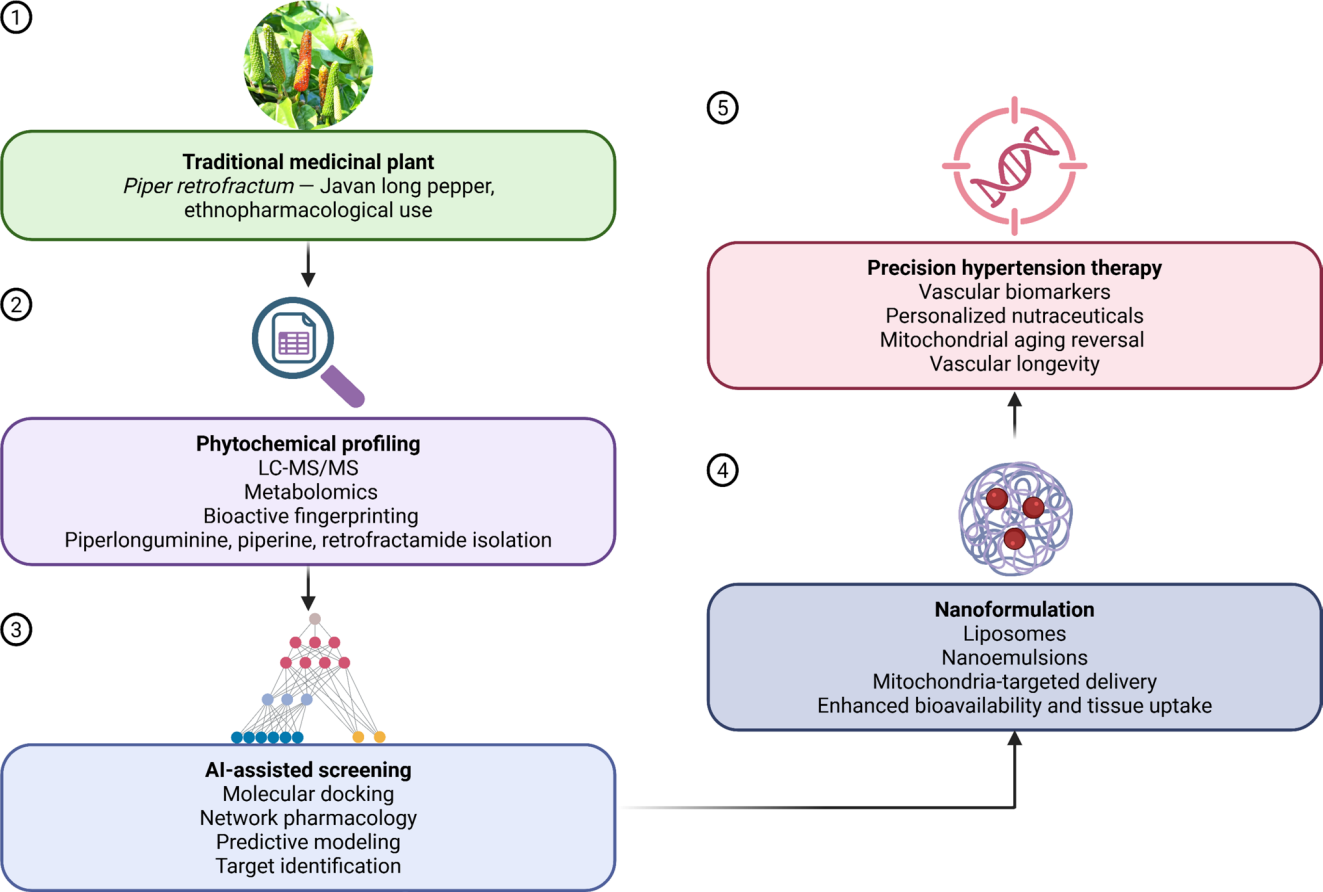
Future translational roadmap for Piper retrofractum-based mitochondrial nutraceuticals in hypertension. Advanced metabolomics, network pharmacology, molecular docking, and artificial intelligence-assisted phytochemical optimization may accelerate identification of bioactive mitochondrial modulators from Piper retrofractum. Integration with nanoformulation strategies and precision vascular biomarkers could facilitate development of personalized nutraceutical interventions targeting mitochondrial dysfunction and vascular aging in hypertensive patients. Disclaimer: This figure is intended as a forward-looking conceptual roadmap and does not represent evidence-established therapeutic or translational pathways; several proposed directions remain speculative and require substantial experimental validation. This figure was created using BioRender.com under a premium license by Fahrul Nurkolis

Looking ahead, several emerging trends are expected to shape future research and applications of *Piper retrofractum* (Fig. 4). One promising direction is the development of precision mitochondrial nutraceuticals, where advances in genetic screening, including mitochondrial DNA haplotypes and polymorphisms in genes such as *SIRT3* and *AMPK*, may enable personalized recommendations of *P. retrofractum*-based interventions for individuals who are most responsive to mitochondrial enhancement therapies. In parallel, artificial intelligence and machine learning approaches may facilitate phytochemical optimization by designing improved analogs of piperine and related Piper amides with enhanced bioactivity, stability, and solubility. AI-driven screening systems could also identify synergistic phytochemical combinations and predict potential herb–drug interactions more accurately [87].

Another important avenue is the development of personalized hypertension therapies based on patient-specific phenotypes and biomarker profiles. Rather than using generalized herbal supplements, future *P. retrofractum* formulations may be tailored to target particular pathological conditions, such as oxidative stress, endothelial dysfunction, or metabolic dysregulation. Advances in mitochondrial biomarker technology, including measurements of circulating cell-free mitochondrial DNA, mitochondrial enzymes, and oxidative stress markers, may further improve the monitoring of therapeutic responses to Piper-based nutraceuticals. For example, reductions in mitochondrial reactive oxygen species (mtROS) markers or increases in mitochondrial coenzyme activity following supplementation could serve as indicators of efficacy [88].

In addition to its therapeutic potential for hypertension, *P. retrofractum* may also be explored for broader vascular longevity and healthy aging applications. Since vascular aging contributes significantly to cardiovascular and metabolic diseases, long-term supplementation studies could investigate whether the plant’s bioactive compounds help slow arterial stiffening and preserve endothelial function in aging populations. Regulatory developments are also likely to play an important role in future translation. As scientific evidence accumulates, clearer regulatory frameworks for mitochondria-targeted botanical therapeutics may emerge, supported by closer collaboration between researchers, industry stakeholders, and public health authorities.

Overall, *P. retrofractum* represents a promising platform for innovation, ranging from molecular engineering of its alkaloids to systems-based modeling of vascular and mitochondrial health. Its potential integration into precision nutrition and personalized cardiovascular prevention strategies highlights an exciting frontier for future biomedical and nutraceutical research.

## Conclusion

*Piper retrofractum* represents a biologically plausible and promising preclinical candidate for mitochondrial-targeted vascular protection in hypertension due to the antioxidant, anti-inflammatory, and metabolic regulatory properties of its major bioactive compounds. Available evidence suggests that piperine, piplartine, retrofractamides, and related phytochemicals may influence pathways associated with mitochondrial oxidative stress, endothelial dysfunction, inflammatory signaling, and vascular bioenergetics. However, much of the current mechanistic support remains indirect and is frequently derived from nonvascular systems, isolated compounds, related *Piper* species, or disease models unrelated to hypertensive vascular mitochondria. Consequently, while the available literature supports mechanistic plausibility, the evidence base remains insufficient to establish *P. retrofractum* as a validated mitochondria-centered antihypertensive intervention.

Several important translational steps are required before any clinical application can be reasonably considered. These include rigorous phytochemical standardization and extract characterization, direct vascular mitochondrial assays in human primary endothelial and vascular smooth muscle cells, dose–response studies in hypertension-specific animal models, comprehensive pharmacokinetic and biodistribution investigations, chronic toxicological evaluations, and assessment of herb–drug interactions with commonly prescribed antihypertensive therapies. Collectively, such studies are essential to determine whether mechanistic promise can translate into clinically meaningful vascular benefit. At present, clinical translation of *Piper retrofractum* for hypertension management remains premature and requires substantially stronger experimental validation.

## Data Availability

No datasets were generated or analysed during the current study.
